# Peptides from *Colochirus robustus* Enhance Immune Function via Activating CD3ζ- and ZAP-70-Mediated Signaling in C57BL/6 Mice

**DOI:** 10.3390/ijms18102110

**Published:** 2017-10-08

**Authors:** Xiaogang Du, Fangliang Lian, Yunkun Li, Dong Li, Dayong Wu, Qunli Feng, Zhijiang Feng, Yun Li, Guixian Bu, Fengyan Meng, Xiaohan Cao, Zhiyu Chen, Xianyin Zeng

**Affiliations:** 1 Department of Engineering and Applied Biology, College of Life Science, Sichuan Agricultural University, Ya’an 625014, China; 18283584303@163.com (F.L.); mapcase895284@163.com (Y.L.); m13540665161@163.com (D.L.); jl15058859543@163.com (Q.F.); guixianbu@sicau.edu.cn (G.B.); 13348895608@163.com (F.M.); 15902804169@163.com (X.C.); wujiayu_0623@163.com (Z.C.); 2 Nutritional Immunology Laboratory, Jean Mayer USDA Human Nutrition Research Center on Aging at Tufts University, Boston, MA 02111, USA; dayong.wu@tufts.edu; 3 Ningbo Yunmi Biological Science and Technology Co., Ltd., Ningbo 315613, China; nbczh2001@163.com (Z.F.); 18980617348@163.com (Y.L.); 4 Ningbo Bofeng Biological Science and Technology Co., Ltd., Ningbo 315613, China

**Keywords:** *Colochirus robustus*, peptides, immune function, CD3ζ, ZAP-70

## Abstract

*Colochirus robustus*, a species of sea cucumber, has long been used in East and Southeast Asia as nutritious food as well as for certain medicinal purpose. Studies have shown a number of biological functions associated with consumption of sea cucumber, many of which are attributed to its major component, sea cucumber peptides (SCP). However, how SCP impacts immune system, which is critical for host defense, has not been defined. To address this issue, in the present study, we conducted comprehensive analysis of immune function after oral administration of SCP (0, 25, 50 and 75 mg/kg body weigh) for eight weeks in C57BL/6 mice. We found that SCP treatment significantly enhanced lymphocyte proliferation, serum albumin (ALB) levels, and the natural killer (NK) cell activity. Moreover, SCP promoted functions of helper T cells (Th) as indicated by increased production of Th1 type cytokines of Interleukin (IL)-1β, IL-2, Interferon (IFN)-γ and TNF-α and Th2 type cytokines (IL-4, IL-6 and IL-10). To determine the effective components, SCP was hydrolyzed into 16 types of constituent amino acids in simulated gastrointestinal digestion and these hydrolytic amino acids (HAA) were used for the mechanistic studies in the in vitro models. Results showed that HAA enhanced lymphocyte proliferation and production of IL-2, IL-10 and IFN-γ. Furthermore, CD3ζ (CD3ζ) and ζ-chain-associated protein kinase 70 (ZAP-70), the signaling molecules essential for activating T lymphocytes, were significantly up-regulated after HAA treatment. In summary, our results suggest that SCP is effective in enhancing immune function by activating T cells via impacting CD3ζ- and ZAP-70-mediated signaling pathway.

## 1. Introduction

Sea cucumber (*Colochirus robustus*), which belongs to *Echinodermata*, *Holothuroidea*, *Aspidochirotida*, has long been used as a tasty, nutritious food as well as a medicinal remedy in some Asian countries (China, Korea and Japan) because of their various health benefits [1,2]. Sea cucumber has been known to improve wound healing and reduce arthritis pain, thus it is widely used in folk medicine for many centuries in China [3,4]. Previous studies have demonstrated that sea cucumber has several bioactivities, such as anti-microbial, anti-oxidant, anti-cancer, anti-angiogenic, anti-hypertension anti-coagulant, and anti-inflammatory functions [5,6]. These proposed therapeutic properties and potential health benefits of sea cucumbers can be attributed to the presence of bioactive compounds including vitamins, minerals, cerebrocides, peptides, and lectins, as well as some unique molecules such as chondroitin sulfates, polysaccharides, sterols, cerebrosides, and saponins [7]. Thus far, however, little attention has focused on the bioactivities of the constituent compounds in sea cucumber, especially for the peptides.

Peptides originated from food proteins can be developed into nutraceuticals which are natural and safe alternatives to synthetic drugs [8]. Peptides, containing 3–20 amino acids in length, form protein primary structure with molecular weight distribution at 100–2000 Da [9]. Multiple biological properties of peptides have been reported, which include anti-microbial and anti-oxidant activities, and as angiotensin-converting enzyme (ACE) inhibitors [10]. Peptides extracted from scorpion venom are effective in recovering immuno-surveillance and intervening immune escape of lung cancer through multi-pathway [11]. Moreover, it has been reported that the peptides from *Pleurotus eryngii mycelium* may be a potential functional food with immunomodulation activity [12]. Recently, Song et al. [13] demonstrated that SCP exerted anti-inflammatory function through inhibiting NF-κB and MAPK activation and inducing HO-1 expression in macrophages. While these results suggest that SCP may modulate innate immune cell functions, it is still elusive as for whether SCP can impact functions of specific immune responses, the more efficient arm of immune system.

A host’s specific immune responses to pathogens include both cellular and humoral immunity. The humoral immune response is induced by B cells and cell-mediated immune defense by T cells [14,15]. It is generally known that plant lectin Concanavalin A (Con A) or T cell receptor (TCR) antibodies anti-CD3/CD28 stimulate T cell proliferation, whereas bacterial endotoxin lipopolysaccharide (LPS) stimulates B cell proliferation. Although many cell types participate in immunoregulation, Th lymphocytes play a critical role in regulating immune responses. Th cells can be further classified into several subsets, including Th1 and Th2, according to differences in their corresponding cytokine expression profiles [16]. Upon TCR stimulation, the ζ-chain interacts with the Src-family tyrosine kinases Lck and Fyn, becomes phosphorylated on its immunoreceptor tyrosine-based activation motifs (ITAM), and recruits the Syk-family protein tyrosine kinase (PTK) ZAP-70 [17]. The most important member of the CD3 family is CD3ζ, to which ZAP-70 binds. CD3ζ and ZAP-70 can facilitate the lymphocyte to proliferate and secrete cytokines.

Given all this, in this study, after we defined the effect of SCP from *Colochirus robustus* on T cell effector functions in the in vivo model, we further investigated effect of SCP on T cell activation with a focus on signaling molecules CD3ζ and ZAP-70 in the in vitro model to help understand the working mechanism of SCP.

## 2. Results

### 2.1. Effect of Sea Cucumber Polypeptides (SCP) on the Body Weight

**Figure 1:**
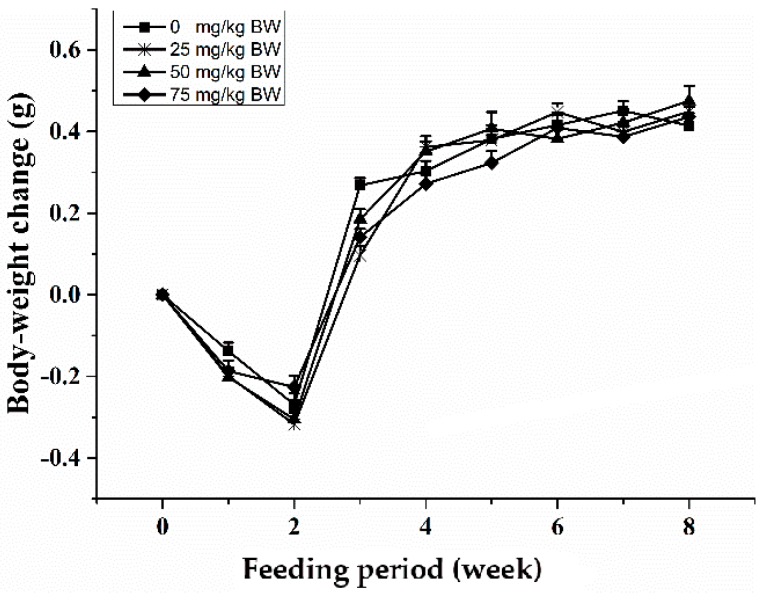
Body-weight change of C57BL/6 mice treated with oral administration of sea cucumber polypeptides (SCP). Mice received daily oral gavage of SCP at 0, 25, 50 or 75 mg/kg body weight for eight weeks. Values are means ± SD, *n* = 10. There was no a significant between control group and SCP group by repeated measures ANOVA (*p* < 0.05). BW, body weight.

Oral gavage of SCP was well tolerated by mice and no abnormal behavior and side effects were observed throughout the experiment. During the first two weeks of the study, animals in all groups had a slight weight loss of 0.2–0.3 g, probably due to the intragastric excitability (Figure 1). After that, body weight steadily increased throughout the study with a comparable rate across all groups so that no treatment-related difference was observed.

### 2.2. Molecular Weight Distribution and Amino Acid Composition

As shown in Table 1, molecular weight distribution of SCP ranged from 100 to 2000 Da (94%) (Table 1). Analysis of amino acid composition of SCP indicated that glycine was the most abundant amino acid present in SCP (18.54 g per 100 g protein), followed by glutamic acid, alanine, arginine, and aspartic acid, accounting for 11.23, 9.75, 7.55 and 6.92 g per 100 g protein, respectively (Table 2).

### 2.3. Effect of SCP on Cellular Composition of Spleen

Compared to the control, mice treated with 75 mg/kg SCP showed a significant increase in percent CD4^+^ and CD8^+^ cells, and mice treated with SCP 50 or 75 mg/kg also showed a higher percent B cells (CD45R^+^) and NK cells (NK 1.1^+^) (Table 3). There was no significant difference in percent regulatory T cells (CD4^+^/CD25^+^) between mice treated with SCP and the control.

### 2.4. Effect of SCP on Lymphocyte Proliferation and Serum Albumin (ALB) Levels

**Figure 2:**
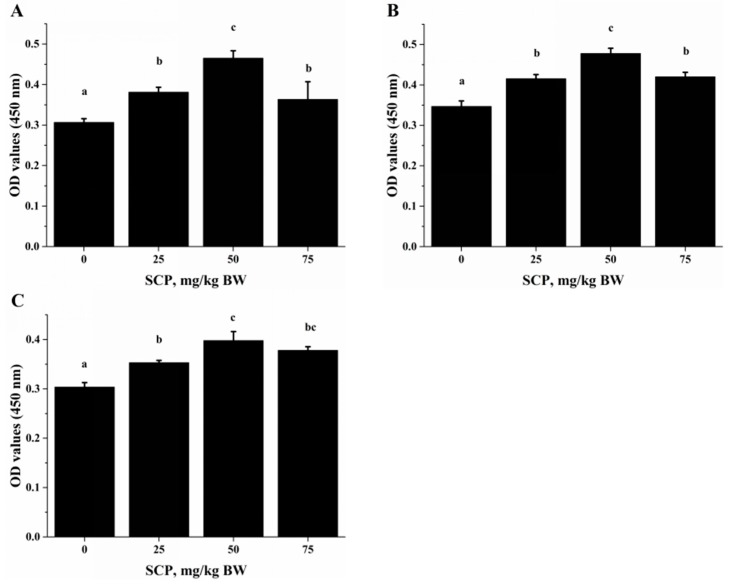
Effect of oral SCP administration on lymphocyte proliferation. C57BL/6 mice treated with SCP at 0, 25, 50 or 75 mg/kg body weight for eight weeks. Splenocytes isolated from these mice were stimulated with: CD3/CD28 (**A**); Concanavalin A (Con A) (**B**); or lipopolysaccharide (LPS) (**C**) for 72 h, and cell proliferation was quantified. Values are means ± SD, *n* = 10. For each variable, means in a row without a common letter significantly differ as determined by one-factor ANOVA, *p* < 0.05. BW: body weight.

**Figure 3:**
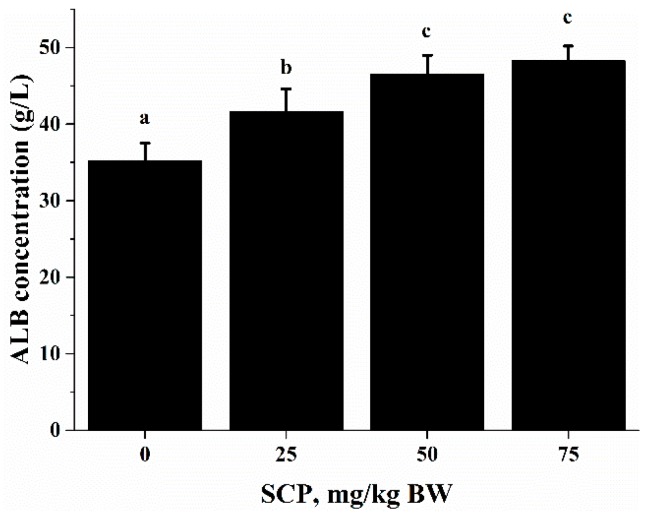
Effect of SCP on ALB concentration in C57BL/6 mice. Mice received daily oral gavage of SCP at 0, 25, 50 or 75 mg/kg body weight for eight weeks. Serum ALB was measured by enzyme linked immunosorbent assay (ELISA) kits. Values are means ± SD, *n* = 10. For each variable, means in a row without a common letter significantly differ as determined by one-factor ANOVA, *p* < 0.05.

Anti-CD3/CD28-induced lymphocyte proliferation was significantly enhanced in mice treated with SCP at all doses (25, 50, 75 mg/kg) with highest increase seen in those receiving 50 mg/kg SCP (*p* < 0.05) (Figure 2A). A very similar pattern of enhancement related to SCP treatment was also observed in the proliferative response of lymphocytes induced by T cell mitogen Con A (*p* < 0.05) (Figure 2B). We also found that SCP treatment enhanced B cell proliferation elicited by B cell mitogen LPS in a dose-dependent pattern up to 50 mg/kg, after which the increase leveled off (*p* < 0.05) (Figure 2C). Serum ALB concentrations were higher in a dose-dependent manner in SCP-treated groups compared to the control group (Figure 3).

### 2.5. Effect of SCP on Natural Killer (NK) Cell Activity

**Figure 4:**
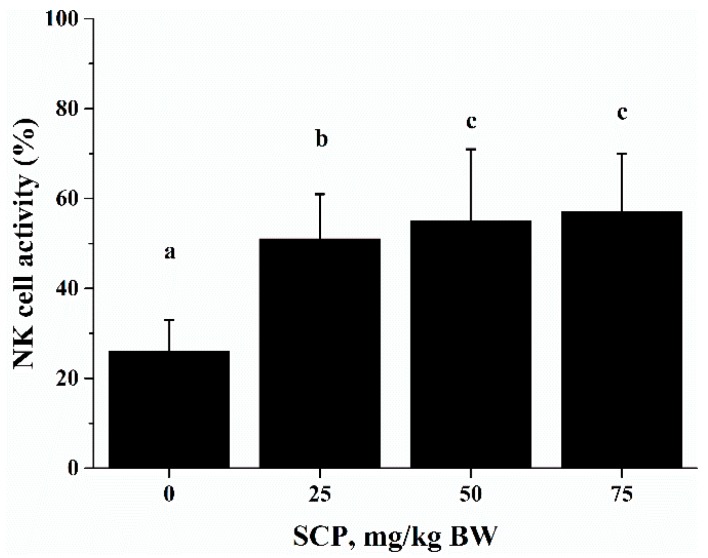
Effect of SCP on natural killer (NK) cells activity in C57BL/6 mice. Mice received daily oral gavage of SCP at 0, 25, 50 or 75 mg/kg body weight for eight weeks. NK activity was determined as percent cytolytic killing of K562 cells (target cells) by splenocytes (effector cells) using a flow cytometry method. Values are means ± SD, *n* = 10. For each variable, means in a row without a common letter significantly differ as determined by one-factor ANOVA, *p* < 0.05.

The cytotoxic activity of NK cells takes part in tumor cell elimination. The cytotoxic activity of splenocytes against NK cell-sensitive K562 cells was measured using an Accuri C6 flow cytometer. The cytotoxic activity of SCP-treated mice was significantly higher than that of phosphate buffer solution (PBS)-treated mice in a dose-dependent manner (*p* < 0.05) (Figure 4).

### 2.6. Effect of SCP on Cytokine Production

Overall, SCP-treated mice had higher CD3/CD28-stimulated production of IL-2, IL-4, IL-6 and TNF-α than those in the control group (Table 4), while IFN-γ was higher only in high dose of SCP treatment (75 mg/kg BW). In addition, higher cytokine production was found in Con A-stimulated cultures from SCP-treated mice compared to the control mice, and it appeared that the effect of SCP was more pronounced in 50 mg/kg BW than in lower (25 mg/kg BW) or higher (75 mg/kg BW) group (Table 5). LPS-stimulated production of IL-1β, IL-6 and TNF-α was higher in SCP groups with similar dose-related pattern as seen in Con A-stimulated cultures (Table 6).

### 2.7. Effect of Hydrolytic Amino Acids (HAA) on Lymphocyte Proliferation

**Figure 5:**
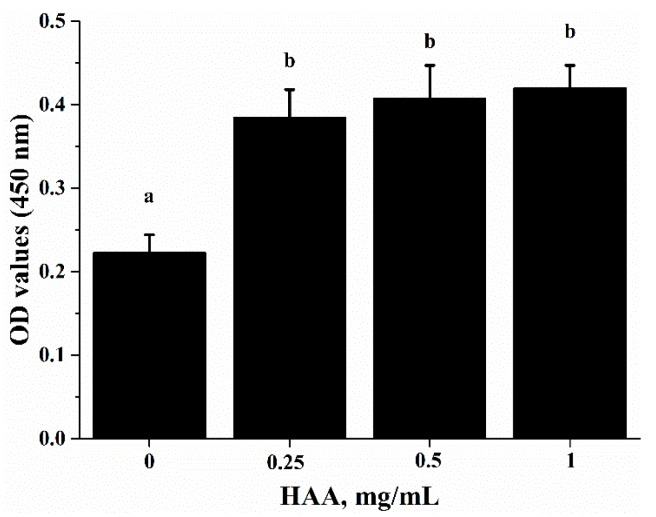
Effect of in vitro HAA supplementation on CD3/CD28-induced lymphocyte proliferation. Splenocytes isolated from C57BL/6 mice were incubated in the presence of HAA at 0, 0.25, 0.5 or 1 mg/mL for 4 h and then cells were stimulated by anti-CD3 (5 mg/mL)/anti-CD28 (1 mg/mL) for 72 h. Cell proliferation was measured by Cell Counting Kit-8(CCK-8) assay. Values are means ± SD, *n* = 10. Means in a row without a common letter significantly differ as determined by one-factor ANOVA, *p* < 0.05.

To verify that SCP-derived HAA (consisted of 16 types of amino acids) contribute to the immuno-enhancing effect of SCP on lymphocyte proliferation, we stimulated splenocytes with anti-CD3/CD28 in the presence of HAA at 0, 0.25, 0.5 and 1 mg/mL. The results showed that HAA significantly enhanced lymphocyte proliferation (Figure 5).

### 2.8. Effect of HAA on Cytokine Production

**Figure 6:**
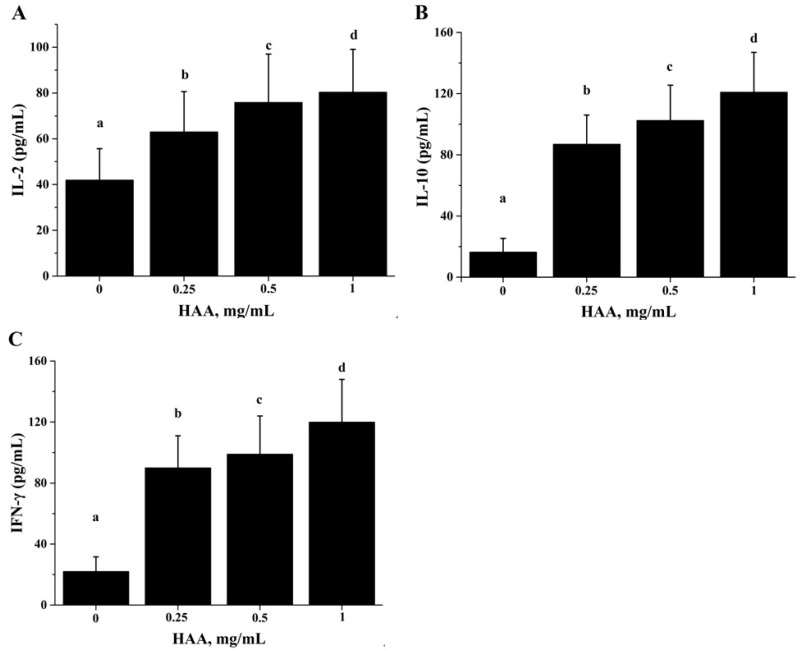
Effect of in vitro HAA supplementation cytokine production. Splenocytes isolated from C57BL/6 mice were incubated in the presence of HAA at 0, 0.25, 0.5 or 1 mg/mL for 4 h and then stimulated by CD3 /CD28 for 48 h. Cell-free supernatant was used to measure production of: Interleukin (IL)-2 (**A**); IL-10 (**B**); and IFN-γ (**C**) by ELISA. Values are means ± SD, *n* = 10. Means in a row without a common letter significantly differ as determined by one-factor ANOVA, *p* < 0.05.

For the same reason, we also determined effect of in vitro HAA supplementation on cytokine production in comparison with SCP. Similarly, we found that HAA enhanced IL-2 (Figure 6A), IL-10 (Figure 6B) and IFN-γ (Figure 6C) production in splenocytes stimulated with anti-CD3/CD28.

### 2.9. Effect of HAA on CD3ζ and ζ-Chain-Associated Protein Kinase 70 (ZAP-70) Expressions

**Figure 7:**
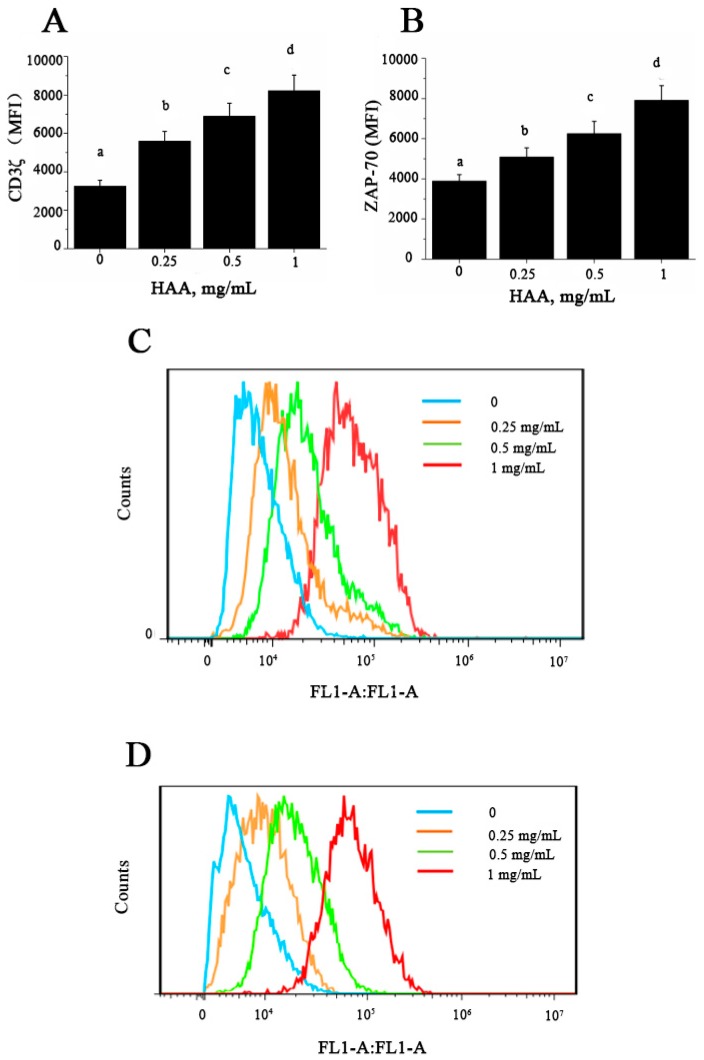
Effect of in vitro HAA supplementation on expression of: CD3ζ (**A**) and ζ -chain-associated protein kinase 70 (ZAP-70) (**B**) Splenocytes were incubated in the presence of HAA at 0, 0.25, 0.5 or 1 mg/mL for 4 h and then stimulated by CD3/CD28 for 48 h. CD3ζ and ZAP-70 expression was determined using flow cytometry; (**A**,**B**) Statistical summary of CD3ζ and ZAP-70 expression presented as mean fluorescence intensity (MFI), respectively; (**C**,**D**) Representative histograms for CD3ζ and ZAP-70, respectively. Values are means ± SD, *n* = 10. Means in a row without a common letter significantly differ as determined by one-factor ANOVA, *p* < 0.05.

CD3ζ and ZAP-70 expressions in T cells are essential steps and thus are used as relevant indicators for T cell activation. To determine whether HAA-induced enhancement in T cell proliferation and cytokine production are related to early activation events in T cells, we tested expression of CD3ζ (Figure 7A,C) and ZAP-70 (Figure 7B,D) in splenocytes stimulated by anti-CD3/CD28 in the presence of HAA. The results indicated that HAA significantly upregulated CD3ζ and ZAP-70 expression.

## 3. Discussion

Previous studies demonstrated that SCP, a 100–2000 Da biological compound, has a wide spectrum of biological effects, including ACE-inhibitory [8], anti-hypertensive [18,19], and antioxidant activities [20]. While very limited information has suggested that SCP may possess bioactivity in modulating immune function [21], there is a lack of comprehensive verification about this and, in particular, the working mechanism for the proposed immuno-stimulatory properties of SCP has not been well elucidated. In this study, we demonstrated that oral administration of SCP increased serum albumin concentrations, lymphocyte proliferation, NK cell activity, and cytokine production, which may be associated with upregulated signaling of CD3ζ and ZAP-70 as indicated in the in vitro mechanistic experiments. These results suggest that SCP may have a potential of serving as a nutraceutical to improve immune system functions.

Lymphocyte proliferation is one of the effective immune responses of T- and B-lymphocytes upon stimulation (such as infection). It has been shown that acidic or neutral peptide fractions stimulated lymphocyte proliferation [22]. In the in vivo study, we robustly stimulated T lymphocyte proliferation with anti-CD3/CD28 (Figure 2A) and Con A (Figure 2B), and B lymphocyte proliferation with LPS (Figure 2C). T-cell-mediated immune response is indispensable for intracellular, in particular the Th cells-derived cytokines that are thought to play a key role in immune function [23]. Th1 type cells are responsible for cell-mediated immune response, while Th2 type cells promote humoral response [24]. The functions of these subsets of Th cells are defined by the cytokines they predominantly produce, for example, IL-2, IFN-γ and TNF-α by Th1 type cells in contrast to IL-4, IL-6 and IL-10 by Th2 type cells [25]. In this study, we found that oral SCP administration increased production of both Th1 and Th2 cytokines. Together, these results suggest that SCP may potentially promote both cellular and humoral immune functions by increasing T cell expansion and secretion of Th1 and Th2 cytokines.

Cytotoxic activity of immune cells is import defense against infectious diseases and cancer [26]. NK cells are a group of specialized cytotoxic lymphocyte characterized by their ability to spontaneously kill tumor cells and virus-infected cells [27,28]. This function of NK cells is mediated and regulated by the immunoregulatory cytokines produced by NK cells themselves as well as other cells such as T cells [29,30]. Consistent with the results of by He et al, who reported that sea cucumber oligopeptides improved NK cell activity [21], in the current study, we observed that oral SCP administration significantly increased NK cell activity. Since we also found an increase in the percentage of NK cells in splenocytes used NK activity assay, it is possible that increased NK activity is largely attributed to increased number of NK cells after SCP treatment. The positive effect of SCP on NK cells suggests that SCP may enhance this innate immune response to potentially prevent viral infection and strengthen the surveillance for tumor development.

ALB is an abundant multifunctional non-glycosylated, negatively charged plasma protein, and its biological functions include ligand-binding and transporting, antioxidant activity, regulating enzymatic activity, and maintaining colloid osmotic pressure and substance metabolism [31]. Health care practitioners have used the ALB level as an index to evaluate nutrition status, specifically protein nutrition status [32]. In this study, we found that oral SCP administration resulted in an elevation in blood ALB concentrations (Figure 3). Deficiency in dietary protein or amino acids is known to impair immune function and increase the susceptibility to infection in both animals and humans. Amino acids are important energy substrates for immune cells, and they are essential for intact functions of immune cells because of their distinct facilitative characteristic [33]. These amino acids include arginine, leucine, isoleucine, valine, glutamine, lysine, threonine, and tryptophan. Increasing evidence have shown that dietary supplementation of specific amino acids to animals and humans with malnutrition and infectious disease can improve their immune status, thereby reducing morbidity and mortality [34]. Arginine supplementation has been reported to enhance T cell response to mitogens [35]. High levels of glutamine, which can result from damaged tissues, modulate lymphocyte proliferation and production of IL-2, IL-10 and IFN-γ in response to stimuli by polarization of the T helper effector response [36,37]. It has been shown that high doses of arginine increase IL-4, IL-10 and TNF-α secretion of T cells, increased concentrations of lysine and leucine promote IL-10 secretion and proliferative activity of T cells, and threonine enhances TNF-α secretion [38]. SCP contains many important amino acids (Table 1), which can be released after SCP is hydrolyzed in a simulated gastrointestinal digestion system in the in vitro study. Using the amino acids (HAA) generated from SCP digestion in the in vitro study, we found that HAA improved the lymphocyte proliferation (Figure 5), and production of IL-2 (Figure 6A), IL-6 (Figure 6B) and IFN-γ (Figure 6C). These results of the in vitro studies further support the results of lymphocyte proliferation (Figure 2) and cytokines production (Table 4) in the in vivo study. Thus, we speculate that SCP may enhance the immune function of mice by increased intestinal absorption of the amino acids derived from SCP.

At present, the underlying mechanism about immunomodulatory effect of SCP remains unclear. Activation of T lymphocytes is induced by binding of MHC-associated peptides with TCR, transduction of CD3-complex, and expression of CD3ζ and ZAP-70 molecules. The T cell receptor ζ chain (CD3ζ) is the principal signal transduction element of the T cell antigen receptor (TCR) [39]. CD8+ T lymphocytes from chagasic donors display reduced proliferative capacity, which might be associated with CD3ζ down-regulation [40]. ZAP-70 is essential for TCR-mediated activation of mature T cells, and it also plays a critical role in T cell maturation. A recent study has demonstrated that deletion of ZAP-70 affects CD2- and CD3-mediated proliferation as well as cytokines production of TNF-α and IFN-γ in T cells [41]. In this study, our results showed that HAA could enhance CD3ζ (Figure 7A) and ZAP-70 (Figure 7B) expression in vitro. T cell proliferation is known to depend on the presence of amino acids in culture and TCR complex expression [42]. Conversely, amino acids depletion causes diminished T cell proliferation, cytokine production, and CD3ζ chain expression [43]. T cells cultured in the absence of amino acids exhibit a sustained down-regulation of CD3ζ preventing the normal expression of TCR, a decreased proliferation, and a significantly diminished production of IFN-γ, IL-5 and IL-10 [44]. Taken together, our results suggest that SCP-induced immuno-enhancement may be because that SCP is digested in the intestine to release its constituent amino acids, which are absorbed into the body and induce up-regulation of CD3ζ and ZAP-70 leading to enhanced T cell proliferation and cytokine production.

## 4. Materials and Methods

### 4.1. Reagents

The body wall of sea cucumber (Colochirus robustus) was obtained from Ningbo Bofeng Biological Science and Technology Co., Ltd. (Ningbo, China). RPMI-1640 medium and fetal bovine serum (FBS) were from Hyclone (Logan, UT, USA). Con A, trypan blue, Dimethyl sulfoxide (DMSO), and LPS were from Sigma (St. Louis, MO, USA). Cell Counting Kit-8(CCK-8) was from Dojindo (Kumamoto, Kyushu, Japan). K562 cell line (Human chronic myelocytic leukemia) was from Bioscience-iCell (Shanghai, China). 5(6)-Carboxyfluorescein diacetate N-succinimidyl ester (CFSE), all primary antibodies, and ELISA kits were purchased from eBioscience (SanDiego, CA, USA).

### 4.2. Preparation of Sea Cucumber Polypeptides (SCP)

SCP was obtained as previously described with some modifications [13]. The fresh body wall of sea cucumber (Colochirus robustus) was rinsed with deionized water. The body wall of sea cucumber was dried and pulverized in order to obtain the powder. The powder was added to PBS and the Flavourzyme of 1% of the body mass of sea cucumber. Then the solution was hydrolysed for 12 h (50 °C, pH 6.8~7.2). The solution was boiled at 90 °C for 10 min to stop enzyme reaction. Subsequently, the solution was added into a 3-fold volume of ethanol solution for 24 h. The supernatant solution of peptides was obtained by centrifugation at 4500 rpm for 25 min. The peptides solution was purified by G10 gel chromatography. Finally, after freeze-dried, SCP was stored at −20 °C until use.

### 4.3. Analysis of Amino Acid Composition and Molecular Weight Distribution

Amino acid composition was measured by an automatic amino acid analyzer following the protocol previously described [45]. The molecular weight distribution of SCP was determined using high performance size exclusion chromatography (HPSEC) as previously described [46]. Briefly, the concentrated SCP dispersion was diluted with 30 volumes (*v*/*v*) of 90% Me2SO, and an aliquot of 50 μL was injected into an HPSEC system with Me2SO as the mobile phase. The raw data were collected using Millennium software and then exported to and processed in MS Excel.

### 4.4. Animal Treatment

Six-week-old male C57BL/6 mice weighed 18–22 g were purchased from the Laboratory Animal Centre at the West China Center of Medical Sciences, Sichuan University (Chengdu, China). After 2-week of acclimation, mice were randomly divided into four groups (10/group) to receive daily gavage of PBS (control), SCP in PBS at 0.25, 0.50 or 0.75 g/kg body weight for 8 week. Mice were individually housed in wire-bottomed cages with free access to drinking water and the AIN-93 diet. Environmentally controlled animal rooms provided a constant temperature at 24 °C, relative humidity at 60–70%, and a 12-h-light/-dark cycle (7:00 am/7:00 pm). All procedures of handling the animals were conformity with the National Institutes of Health (NIH) guidelines (Pub. No. 85-23, revised on 1 September 1996) and was approved by Animal Care and Use Committee of the Sichuan Agricultural University.

### 4.5. Hydrolytic Amino Acids (HAA) Preparation

SCP (100 mg) was added to a hydrolysis tube and then sealed with 50 mL of 6 mol/L HCl solutions. After the hydrolysis tube was incubated at 110 °C in an incubator for 24 h, the solution was concentrated by rotary evaporator to remove HCl solutions. The resulting dried free amino acids were collected with constant volume PBS as HAA to be used for the in vitro study.

### 4.6. Analysis of Splenocyte Phenotype

After mice were sacrificed by CO2 asphyxiation, spleens were aseptically removed and placed in sterile plates containing RPMI 1640. Single cell suspensions were isolated by gently disrupting spleens, and passed through a 200-mesh stainless steel sieve. After red blood cells were removed using red blood cell lysis buffer (8.29 g/L NH4Cl, 1 g/L KHCO_3_ and 37.2 mg/L Na2EDTA), splenocytes were washed twice and then suspended in 1 mL complete RPMI-1640 medium containing 10% (*v*/*v*) FBS, 100 kU/L penicillin and 100 mg/L streptomycin. Cell viability was assessed by the trypan blue exclusion method.

To determine the cellular composition of spleen, 1 × 10^6^ splenocytes were blocked with 0.5 µL Anti-CD16/32 (Fcγblock) (0.5 mg/mL) for 30 min at 4 °C, followed by 3 times of wash with PBS. Splenocytes were then stained in 3 sets of combinations: FITC-conjugated anti-mouse CD3, PE-conjugated anti-mouse CD4 and APC-conjugated anti-mouse CD8 to identify total T cells, CD4+ and CD8+ T cells; APC-conjugated anti-mouse CD45R, FITC-conjugated anti-mouse CD3 and PE-conjugated anti-mouse NK1.1 were used to identify B cells and natural killer cells; FITC-conjugated anti-mouse-CD4 and PE-conjugated anti-mouse-CD25 were used to identify regulatory T cells. Stained cells were analyzed by an Accuri C6 flow cytometer (BD Accuri Cytometers, Ann Arbor, NJ, USA) and acquired data were analyzed using CFlow software (BD Accuri Cytometers, Ann Arbor, NJ, USA).

### 4.7. Lymphocyte Proliferation Assay

The lymphocyte proliferation was performed as previously described [47]. Briefly, splenocytes (1 × 10^5^ cells/well) in 96-well flat-bottom plates (Costar^®^ Assay Plate, Corning Incorporated, Corning, New York, NY, USA) were cultured with or without the presence of T cell mitogen Con A at 1.5 mg/L, LPS at 1 mg/L, or plate-coated anti-CD3 (5 mg/L) plus soluble anti-CD28 (1 mg/L) (CD3/CD28). Plates were incubated for 72 h at 37 °C and 5% CO2. During the last 4 h, 10 μL/well of CCK-8 solution was added to plates. The absorbance was measured at 450 nm using a Synergy HT plate reader (BIO-TEK, Winooski, VT, USA).

For the in vitro lymphocyte proliferation assay, splenocytes isolated from C57BL/6 mice were incubated with HAA at concentrations of 0, 0.25, 0.5 and 1 mg/mL for 4 h before stimulated by anti-CD3 (5 mg/L)/anti-CD28 (1 mg/L) for 72 h. Cell proliferation was measured as OD 570 nm using a plate reader.

### 4.8. Serum Albumin (ALB) Concentration Assay

At the end of the 8-week oral SCP administration, blood samples were collected into EDTA Eppendorf tubes by retro-orbital venous plexus puncture from mice under anesthesia. Blood samples were centrifuged at 2000 rpm for 5 min at 4 °C and obtained serum samples were stored at −80 °C. ALB concentrations were measured using sandwich ELISA kits following the manufacturer’s instructions. The absorbance of the solutions was measured at 450 nm using a plate reader.

### 4.9. Measurement of Cytokines

Splenocytes (1 × 10^6^ cells/well) in 24 well plates were cultured in the presence of Con A (1.5 mg/L) or anti-CD3 (5 mg/L)/anti-CD28 (1 mg/L) for 72 h at 37 °C in 5% CO2 for cytokines IL-2, IL-4, IL-6, IL-10, IFN-γ and TNF-α production, or in the presence of LPS (1 mg/L) for 72 h for IL-1β, IL-6 and TNF-α. Cell-free supernatants were collected at the end of incubation and stored at −20 °C for later analysis. Cytokine production was measured using sandwich ELISA kits following the manufacturer’s instructions.

For the in vitro cytokine production assay, splenocytes were incubated with HAA at 0, 0.25, 0.5 or 1 mg/mL for 4 h before being stimulated by CD3 (5 mg/L)/CD28 (1 mg/L) for 48 h for examining IL-2, IL-10 and IFN-γ production.

### 4.10. Natural Killer (NK) Cell Activity Assay

NK cell activity was determined as previously described with some modifications [14,48]. Briefly, K562 cells as target cells were labeled with CFSE (2.5 µM). The splenocytes were used as the effector cells. Splenocytes and K562 cells were mixed at ration 50:1 (effector: target) in 96-well plates. After mixed cells were incubated for 4 h at 37 °C and 5% CO2, 0.25 µL PI solution (1 mg/mL) was added into each well and incubation continued for additional 10 min. CFSE-stained cells and PI-stained cells were determined by an Accuri C6 flow cytometer. NK cells activity was calculated using the following formula: NK cells activity (%) = [dead K562 Cells (%) − spontaneously dead K562 Cells (%)] × 100/[100 − spontaneously dead K562 target cells (%)].

### 4.11. CD3ζ and ZAP-70 Expression

CD3ζ and ZAP-70 expression was measured using a protocol as previously described [49]. In brief, splenocytes (5 × 10^6^ cells/ml) were pre-incubated with RPMI/10% FBS at 4 °C for 10 min and HAA (0, 0.25, 0.5, 1 mg/mL) was added to incubation for 4 h before stimulated by CD3 (5 mg/L)/CD28 (1 mg/L) for 48 h. Cells were stained with PerCP-Cy5.5 conjugated anti-CD3 for 30 min at 4 °C. After washed 3 times, cells were incubated in permeabilization buffer for 10 min. Cells were then re-suspended in PBS, and stained with anti-CD3ζ-FITC or anti-ZAP-70-FITC antibody at 4 °C for 2 h, followed by washing 5 times before analysis. Analysis was performed using CFlow software and expression levels of CD3ζ and ZAP-70 were measured as mean fluorescence intensity (MFI) and percent of positive cells.

### 4.12. Statistical Analysis

All results were expressed as the mean ± standard deviation (S.D.). Statistical analysis was conducted using SPSS software version 23.0 (SPSS Inc., Chicago, IL, USA). One-factor analysis of variance (ANOVA) was used to analyze the data. Values with *p* < 0.05 were considered statistically significant.

## 5. Conclusions

In summary, in this study, we have shown that oral SCP administration can enhance immune response in mice. This effect of SCP may be associated with increased intake of SCP-derived amino acids which upregulate signaling pathways involving CD3ζ and ZAP-70 activation. These results suggest that SCP has a promising potential as a functional food to improve body’s immune function and resistance to infection. Future studies are needed to confirm these findings and, more importantly, to determine its translational value in disease prevention and application in humans. On the other hand, the mechanistic study presented here represents only a preliminary attempt and further expansion to this end is warranted.

## Figures and Tables

**Table 1:** Molecular weight distribution of Sea Cucumber Polypeptides (SCP).

Molecular Weight Range	%
<100	1.50
100~300	28.50
300~600	30.00
600~1000	20.00
1000~2000	15.50
2000~3000	3.00
>3000	1.50

**Table 2:** Amino acid composition of SCP.

Amino Acid	g/100 g Protein
Gly	18.54
Glu	11.23
Ala	9.75
Arg	7.55
Asp	6.92
Pro	5.90
Thr	4.68
Ser	4.50
Leu	4.39
Val	3.83
Lys	3.33
Ile	2.29
Tyr	2.10
Phe	1.80
Met	1.77
His	1.57

**Table 3:** Effect of oral administration with the SCP on lymphocyte cell phenotype of C57BL/6 mice.

Group (mg/kg BW)	CD4^+^ T Cells (%)	CD8^+^ T Cells (%)	B Cells (%)	NK (%)	Treg (%)
0	29.24 ± 3.32 ^b^	15.61 ± 2.91 ^b^	55.12 ± 3.71 ^a^	2.23 ± 0.46 ^a^	3.34 ± 0.68 ^a^
25	28.11 ± 3.12 ^a^	14.96 ± 2.37 ^a^	55.52 ± 4.34 ^a^	2.41 ± 0.62 ^a^	3.21 ± 0.53 ^a^
50	29.55 ± 3.73 ^b^	15.37 ± 2.14 ^ab^	56.78 ± 5.16 ^b^	2.96 ± 0.77 ^b^	3.28 ± 0.76 ^a^
75	30.47 ± 4.21 ^c^	16.16 ± 2.74 ^c^	56.43 ± 4.83 ^b^	3.37 ± 0.85 ^c^	3.14 ± 0.52 ^a^

Values are means ± SD, *n* = 10. For each variable, means in a row without a common letter differ by one-factor ANOVA, *p* < 0.05. NK: natural killer; BW: body weight.

**Table 4:** Effect of oral administration with the SCP on CD3/CD28-induced cytokines production by lymphocyte of C57BL/6 mice.

Group (mg/kg BW)	IL-2 pg/mL	IL-4 pg/mL	IL-6 pg/mL	IL-10 pg/mL	TNFα pg/mL	IFNγ pg/mL
0	310.4 ± 28.1 ^a^	62.3 ± 6.1 ^a^	44.5 ± 3.8 ^a^	425.3 ± 51.2 ^c^	215.7 ± 16.5 ^b^	204.2 ± 17.1 ^c^
25	412.8 ± 32.7 ^b^	110.5 ± 8.2 ^b^	61.5 ± 5.5 ^c^	386.4 ± 32.5 ^a^	228.1 ± 18.2 ^c^	140.6 ± 10.7 ^a^
50	488.1 ± 25.7 ^c^	130.1 ± 7.9 ^c^	65.4 ± 4.7 ^d^	414.9 ± 49.1 ^b^	240.1 ± 27.3 ^d^	157.4 ± 16.3 ^b^
75	400.2 ± 34.1 ^b^	100.9 ± 5.6 ^b^	50.3 ± 4.1 ^b^	429.5 ± 46.4 ^c^	203.6 ± 13.2 ^a^	228.3 ± 25.2 ^d^

Values are means ± SD, *n* = 10. For each variable, means in a row without a common letter differ by one-factor ANOVA, *p* < 0.05. IFN: Interferon.

**Table 5:** Effect of oral administration with the SCP on Concanavalin A (Con A)-induced cytokines production by lymphocyte of C57BL/6 mice.

Group (mg/kg BW)	IL-2 pg/mL	IL-4 pg/mL	IL-6 pg/mL	IL-10 pg/mL	TNFα pg/mL	IFNγ pg/mL
0	182.2 ± 25.3 ^b^	12.1 ± 0.9 ^a^	26.3 ± 2.1 ^b^	70.9 ± 5.6 ^a^	551.5 ± 22.4 ^a^	58.3 ± 4.3 ^a^
25	157.6 ± 19.1 ^a^	16.3 ± 1.8 ^c^	22.1 ± 1.3 ^a^	72.4 ± 3.2 ^a^	575.1 ± 31.6 ^b^	72.2 ± 5.1 ^b^
50	207.3 ± 32.4 ^c^	16.6 ± 1.6 ^c^	30.2 ± 3.1 ^c^	125.5 ± 10.8 ^c^	641.4 ± 56.3 ^d^	100.2 ± 15.2 ^d^
75	189.5 ± 23.6 ^b^	13.3 ± 1.4 ^b^	31.1 ± 2.5 ^c^	80.2 ± 7.5 ^b^	592.3 ± 41.1 ^c^	84.4 ± 8.6 ^c^

Values are means ± SD, *n* = 10. For each variable, means in a row without a common letter differ by one-factor ANOVA, *p* < 0.05.

**Table 6:** Effect of oral administration with the SCP on lipopolysaccharide (LPS)-induced cytokines production by lymphocyte of C57BL/6 mice.

Group (mg/kg BW)	IL-1β pg/mL	IL-6 pg/mL	TNFα pg/mL
0	129.2 ± 10.1 ^a^	53.2 ± 3.8 ^a^	140.6 ± 34.3 ^a^
25	134.5 ± 17.3 ^a^	61.7 ± 5.8 ^c^	159.9 ± 51.2 ^c^
50	157.8 ± 26.2 ^b^	67.7 ± 8.1 ^d^	202.3 ± 62.5 ^d^
75	131.1 ± 12.7 ^a^	56.2 ± 4.9 ^b^	149.4 ± 44.3 ^b^

Values are means ± SD, *n* = 10. For each variable, means in a row without a common letter differ by one-factor ANOVA, *p* < 0.05.
